# Eco-friendly *p*-type Cu_2_SnS_3_ thermoelectric material: crystal structure and transport properties

**DOI:** 10.1038/srep32501

**Published:** 2016-09-26

**Authors:** Yawei Shen, Chao Li, Rong Huang, Ruoming Tian, Yang Ye, Lin Pan, Kunihito Koumoto, Ruizhi Zhang, Chunlei Wan, Yifeng Wang

**Affiliations:** 1 Nanjing Tech University, College of Materials Science and Engineering, Nanjing 210009, China; 2 East China Normal University, Key Laboratory of Polar Materials and Devices, Shanghai 200062, China; 3 Toyota Physical and Chemical Research Institute, Nagakute 480-1192, Japan; 4 Jiangsu Collaborative Innovation Center for Advanced Inorganic Function Composites, Nanjing Tech University, Nanjing 210009, China; 5 Northwest University, Department of Physics, Xi’an 710069, China; 6 Tsinghua University, School of Materials Science and Engineering, Beijing 100084, China

## Abstract

As a new eco-friendly thermoelectric material, copper tin sulfide (Cu_2_SnS_3_) ceramics were experimentally studied by Zn-doping. Excellent electrical transport properties were obtained by virtue of 3-dimensionally conductive network for holes, which are less affected by the coexistence of cubic and tetragonal phases that formed upon Zn subsitition for Sn; a highest power factors ~0.84 mW m^−1^ K^−2^ at 723 K was achieved in the 20% doped sample. Moreover, an ultralow lattice thermal conductivity close to theoretical minimum was observed in these samples, which could be related to the disordering of atoms in the coexisting cubic and tetragonal phases and the interfaces. Thanks to the phonon-glass-electron-crystal features, a maximum *ZT* ~ 0.58 was obtained at 723 K, which stands among the tops for sulfide thermoelectrics at the same temperature.

Eco-friendly sulfides have attracted special interests beyond telluride and selenides in various fields including thermoelectrics (TEs) for which a high *ZT* value termed as thermoelectric dimensionless figure of merit is desired for practical applications of power generation and steady-state cooling (*ZT* = *S*^2^*σT*/*κ*, where *S* is Seebeck coefficient, *σ* is electrical conductivity, *T* is absolute temperature, and *κ* is thermal conductivity comprising of electronic component *κ*_*e*_ and phonon component *κ*_*lat*_)^1^. Especially encouraging are the recent findings in binary copper sulfide of Cu_1.8_S^2^ with *ZT* ~ 0.5 at 673 K and superionic α-Cu_1.97_S with phonon-liquid-electron-crystal (PLEC) behavior^3^ and a strikingly high *ZT* ~ 1.7 at 1000 K. Nevertheless, the liquid-like electromigration of Cu and the phase transitions would be detrimental for its long-term TE performance^4^. Alternatively, researches on multinary sulfides have demonstrated a much higher structural and electrical stability due to the pinning effect of atoms other than Cu, indicating their promising prospect as TEs^5^.

Focused in the present study is a ternary compound of copper tin sulfide, Cu_2_SnS_3_ (hereafter CTS), which has a melting point of 1129 K^6^ and a direct band gap of 0.9–1.3 eV^7^,^8^. CTS has been reported to adapt three different structures as shown in Fig. 1, including monoclinic (ICSD-91762), cubic (ICSD-43532) and tetragonal (ICSD-50965) symmetries that can be viewed as superstructure derivatives from sphalerite type structure. These CTS structures are all based on corner-sharing tetrahedra with Cu and Sn^9^,^10^ positioned at the tetrahedral site coordinated with 4 S atoms at the corners and thus are recognized as diamond-like ternary compounds, but the arrangement of metal atoms inside the S sublattice is different from each other. In the monoclinic structure of CTS, Cu and Sn atoms fully occupy the separated 2*a* tetrahedral sites orderly. While in the tetragonal structure, there are three different occupations for the tetrahedral sites, i.e. the 2*a* tetrahedral sites (accounting for 1/4 of total) are occupied by Cu atoms only, and the 4*d* and 2*b* sites (1/2 and 1/4 of total for each) are occupied by the composite atoms M1 [43.6(2) at% Sn + 56.4(2) at% Cu] and M2 [46.3(3) at% Sn + 53.7(3) at% Cu], respectively, which means a high-degree disordering of Cu and Sn atoms at these sites. For the cubic structure, however, the tetrahedral sites become equivalent and are fully occupied by composite atoms M3 (66.7% Cu + 33.3% Sn), corresponding to a complete disordering of metal atoms in this structure. Besides, the MS_4_ tetrahedra are symmetric only in the cubic structure, while distorted in the monoclinic and tetragonal ones with different M-S distances and M-S-M bond angles. The structural anharmonicity features of atoms disordering and structural distortion would favor in disrupting the phonon transport to suppress the lattice thermal conductivity^10^,^11^,^12^, let alone the effect of interface and boundary in case of multiphase coexistence, which is commonly observed in similar ternary sulfide systems. Moreover, they all possess excellent electrical transport properties due to its 3-dimentional conductive network of hybridized 3*d*(Cu)-3*p*(S) orbitals and S-S 3*p* orbitals at the upper valence band, which is in favor of *p*-type TE performance due to its large density-of-states (DOS)^13^. As a result, the CTS materials can be a potential material of phonon-glass-electron-crystal which is an important characteristic of good TEs. By now, however, little has been reported in particular on CTS as a TE candidate.

Here, we investigated the pristine and hole-doped CTS in form of bulk ceramics with respect to crystal structure and TE properties, for which Zn was selected as the acceptor dopant for substituting Sn in view of its similar radius (60 pm, C.N. = 4) to that of Sn^4+^ (55 pm, C.N. = 4) and Cu^+^ (60 pm, C.N. = 4)^14^. Results revealed a monoclinic-tetragonal and/or cubic crystal structure evolution upon Zn-doping, and a low thermal conductivity was obtained in the heavily doped samples, for which the cation disordering in the derived cubic and tetragonal phases should have played a primary role. Moreover, the method of Zn-doping led to a significant improvement of electrical transport properties, with a maximum power factor of ~0.8 mW m^−1^ K^−2^ at 723 K. And as a result, a highest *ZT* of ~0.58 at 723 K was achieved, which stands among the tops of sulfide TEs, suggesting CTS as a promising eco-friendly TE material.

## Results and Discussion

**Figure 2:**
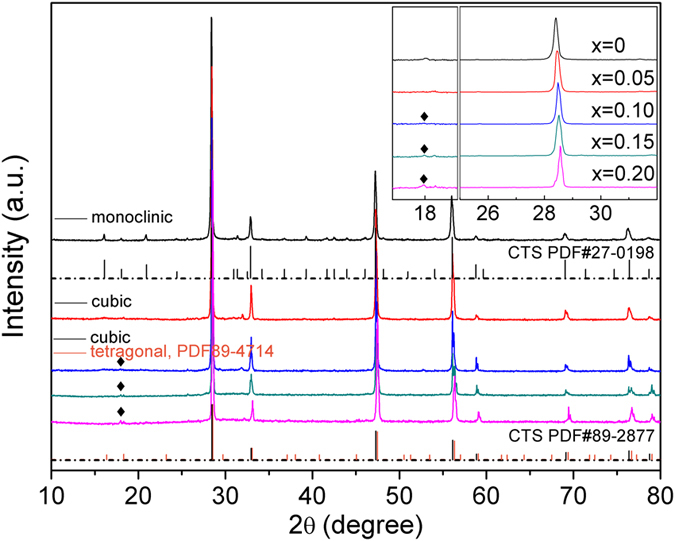
Powder X-ray diffraction patterns of Cu_2_Sn_1−*x*_Zn_*x*_S_3_ samples; the inset shows an enlargement of the low-angle (17–19°, 25–32°) region.

For the sample pellets sintered by spark plasma sintering (SPS) from synthesized powders, the phase composition and crystal structure were analyzed first by powder X-ray diffraction (XRD). Results shown in Fig. 2 revealed an interesting structural evolution in Cu_2_Sn_1−*x*_Zn_*x*_S_3_ with *x* from 0 to 0.20. As one can see for the pristine (*x* = 0) sample, the diffraction patterns are indexed well to the monoclinic structure (PDF#27-0198) in agreement with the prediction by Onoda *et al*.^10^, while different from the report by Chen *et al*.^11^, presumably due to the different synthetic parameters. Upon Zn-doping with *x* = 0.05, Cu_2_Sn_1−*x*_Zn_*x*_S_3_ turned to adopt a cubic structure (PDF#89-2877), which is not the case for the model employed in a previous simulation work^13^ by Zhang *et al*., where a monoclinic symmetry was presumed to be preserved. With *x* further increasing to 0.20, the XRD patterns of Cu_2_Sn_1−*x*_Zn_*x*_S_3_ were found to be similar to that of cubic one, except for some tiny diffraction peaks due to the tetragonal structure (PDF#89-4714), e.g. the (101) peak at 18.31° which was absent in the cubic phases (marked with ♦ in the inset of Fig. 2), suggesting that in these samples the tetragonal phase may coexist as a secondary phase. Besides, a continuous shift of the (111) and (112) peaks respectively for cubic and tetragonal structures at around 28.3° was observed toward the higher 2θ side with *x* increasing from 0.05 to 0.20 as shown in the inset, which indicated a lattice contraction instead of expansion of *a* for the two structures, which should be caused by the further ionization of Cu^+^ into smaller-sized Cu^2+^ as discussed later.

**Figure 3:**
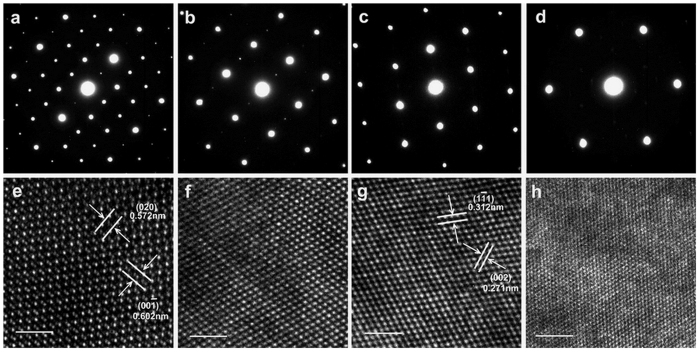
SAED patters of the undoped sample along the zone axis of [100] (**a**), and [110] (**b**), and of the 5% Zn-doped sample along [110] (**c**) and [111] (**d**). (**e**–**h**) are the corresponding HRTEM images of (**a**–**d**), respectively, with a scale bar of 5 nm.

**Figure 4:**
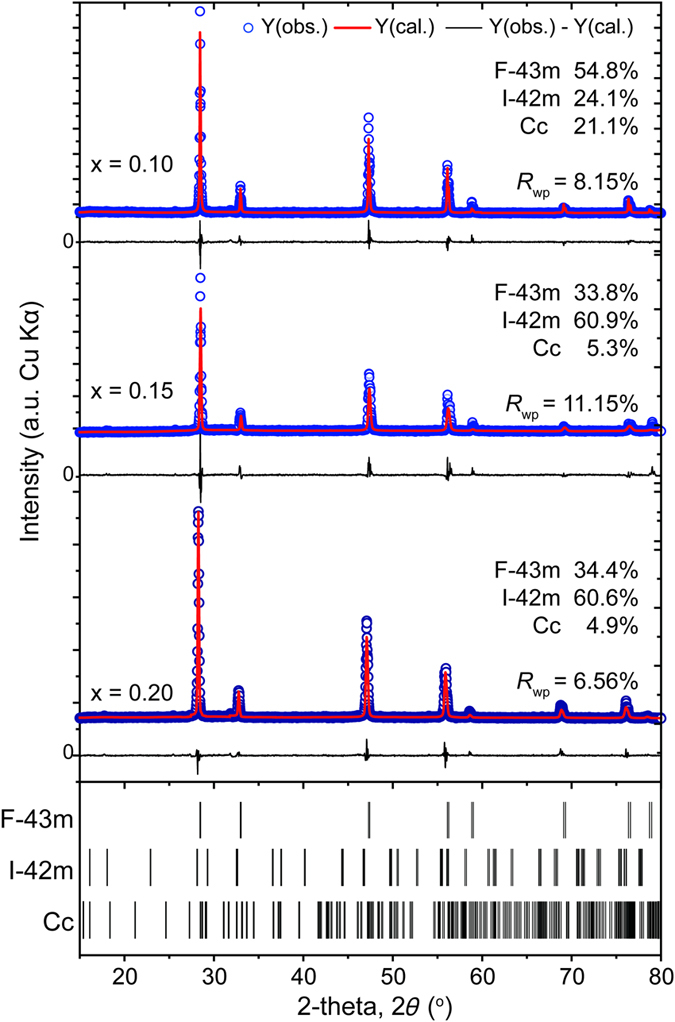
Rietveld refinement results for Cu_2_Sn_1−*x*_Zn_*x*_S_3_ samples with *x* = 0.10–0.20 using Rietan-VENUS program, which deduce the mole ratio of the monoclinic, cubic and tetragonal phases.

In order to confirm the crystal structure evolution corresponding to Zn doping, the Cu_2_Sn_1−*x*_Zn_*x*_S_3_ ceramic samples with *x* = 0 and 0.05 were selected for transmission electron microscopy (TEM) observation. Figure 3a shows a typical selected-area electron diffraction (SAED) pattern of the pristine sample. It can only be indexed as the [100] zone axis with monoclinic symmetry. By tilting the sample about 28.5°, another SAED pattern was obtained, as shown in Fig. 3b, which was indexed as the monoclinic [110] zone axis. The SAED pattern obtained from the 5% Zn-doped sample (Fig. 3c) exhibits typical diffraction patterns of the cubic [110] direction. When tilted about 35.3° to the [111] zone axis, observed is the pattern as shown in Fig. 3d. These careful SAED analyses unambiguously confirmed that the pristine sample took the monoclinic structure whereas the 5% Zn-doped sample took the cubic structure, consistent with the previous XRD analyses. Figures 3e–h are the corresponding high resolution TEM (HRTEM) images of Fig. 3a–d, respectively. The lattice fringes of the monoclinic (020) and (00-1) planes are clearly seen in Fig. 3e, and so are those of the cubic (1–11) and (002) planes in Fig. 3g, demonstrating good crystallinity and high quality of the samples. Considering the possible coexistence of multiphase in Cu_2_Sn_1−*x*_Zn_*x*_S_3_ samples with *x *= 0.10 ~ 0.20, the XRD patterns were analyzed with Rietveld refinement method using program RIETAN-VENUS to deduce the quantitative mass ratio of monoclinic (space group: Cc), orthorhombic (space group: I-42 m) and cubic (space group: F-43 m) phases. The derived index of R_wp_ was low, suggesting good fitting between the experimental and calculated intensities, as shown in Fig. 4. Refinement results (Table 1) show that, with the increase of *x*, the mole fraction of cubic phase decreased from 54.8% (*x *= 0.10) to about 34% (*x *= 0.15, 0.20), with an increasing content of tetragonal phase from 24.1% to 60.6% correspondingly, while that of monoclinic phase was lowered from 21.1% to ~5%. Although the microstructure features (e.g. lattice defects and strains) of the grain boundaries and interfaces are still unknown, the coexistence of these phases should serve to additionally intensify phonon scattering, beside the effect owing to disordered arrangement of metal atoms, to reach a low lattice thermal conductivity. Nevertheless, due to the similarity of 3-dimensionally interconnected conductive network of Cu-S and S-S, the coexistence mainly of cubic and tetragonal structures would not affect the electrical transport properties severely for the doped samples, as discussed in the following contexts.

**Figure 5:**
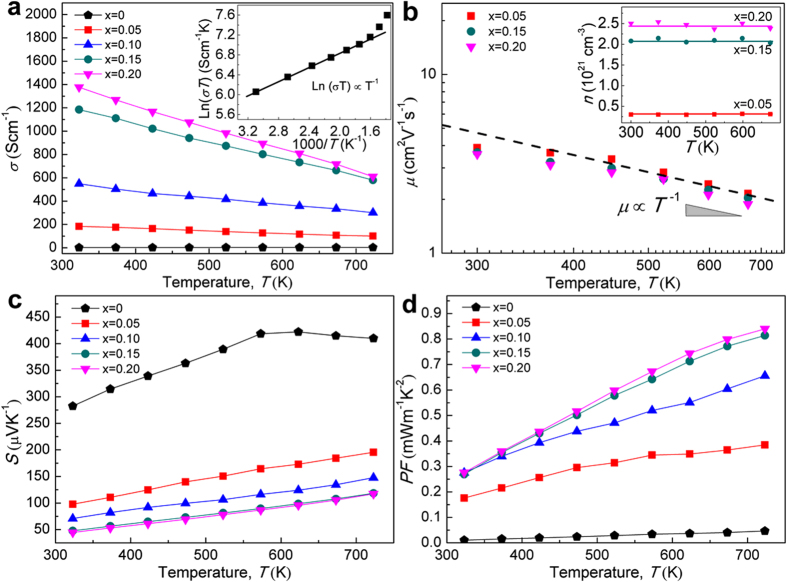
TE properties of the Cu_2_Sn_1−*x*_Zn_*x*_S_3_ samples: (**a**) electrical conductivity, (**b**) hole mobility, (**c**) Seebeck coefficient, (**d**) power factor. The inset in (**a**) shows the electrical conductivity as a function of reciprocal temperature for pristine CTS and the inset in (**b**) shows the carrier concentration. The dash line in (**b**) represents the *μ* ∝ *T*^−1^ relationship.

As to the electrical transport properties, electrical conductivity (*σ*) and Seebeck coefficients (*S*) for all samples are shown in Fig. 5. The pristine CTS exhibits a very low *σ*, indicative of a non-degenerate state with a low carrier concentration. The *σ* value increases gradually with increasing temperature from 323 K to 573 K, following strictly a small polaron hopping model^15^, which reflects the localized states of Cu-3*d* and S-3*p* orbitals when the sample is undoped and thus essentially non-degenerate. The increase of *σ* at temperatures above 573 K should be resulted by the intrinsic thermal bipolaron excitation. By contrast, the *σ* values for the doped compounds decrease with increasing temperature, showing a degenerate semiconductor behavior; Moreover, they increase almost linearly with the amount of Zn. These results can be explained by that the substitution of Zn ([Ar]3*d*^10^4*s*^2^) for Sn ([Kr]4*d*^10^5*s*^2^5*p*^2^) would have introduced acceptor levels within the band gap that trapped the electrons excited from the valence band maximum of the hybridized *d-p* orbitals, forming Cu^2+^ essentially and leaving behind the increased concentration of holes and the metallic electrical properties consequently.

To gain more insight into the transport properties in these CTS compounds, Hall Effect measurement was performed to determine the carrier concentration *n* and its mobility *μ*. As shown in Fig. 5b, *n* of all samples (see the inset) remain essentially constant across the whole temperature range of interest, and they increase with the Zn content, *x*, reaching a high level of 10^21^ cm^−3^. The Hall mobility *μ* decreases proportionally to *T*^−1^ as temperature increases above 448 K, which is characteristic of the predominant acoustic phonon scattering^16^,^17^. The departure below 448 K reveals that impurity scattering from ionized defects becomes important near the room temperature. More importantly, the *μ* values are very close in the doped cubic and cubic-tetragonal mixed phases of CTS, which is caused by that the pathways for holes in these structures are similar, being along Cu-S-Cu channels 3-dimensionally extending throughout the crystal lattices despite the slight difference regarding the coordination of metal atoms in the different phases, and are less affected by the impact of dopant atoms.

Shown in Fig. 5c are the *p*-type Seebeck coefficients (*S*) for Cu_2_Sn_1−*x*_Zn_*x*_S_3_ (*x *= 0–0.20) samples. For the pristine CTS, *S* climbs as temperature rises up to 573 K, the decrease thereafter (see the inset of Fig. 5a) is probably due to the intrinsic thermal excitation of electrons from valence to conduction bands, which is in accordance with the rapid increase of *σ* from 573 K. For all the Zn-doped samples, the *S* values for Cu_2_Sn_1−*x*_Zn_*x*_S_3_ decrease with increasing *x* due to the enhanced *n*, e.g. from 410.0 μV K^−1^ (*x* = 0) to 117.3 μV K^−1^ (*x* = 0.20) at 723 K. The *S* increases almost linearly with increasing temperature due to the continuous increase of chemical potential, while without a perceivable abrupt change which has occurred in Cu_1.8_S^2^, reflecting the preservation of initial crystal structure in the studied temperature range. This phenomenon is considered beneficial to improving the structural durability of TE devices against temperature fluctuation during the long-term operation. The *S* values decrease regularly with increasing *σ*, which seems to suggest that the aforementioned phase change has little influence on *S* in agreement with the deduction that the valence band is almost unaffected by the dopant atoms as discussed above. The *S* data are basically comparable with other representative tetrahedron-based sulfides (Cu_1.8_S^2^, Cu_2_ZnSnS_4_^18^) but are a little smaller than that of the state-of-the-art selenides and telluride TEs with a similar *σ*, e.g., Pb_1−*x*_Na_*x*_Se (~160 μV K^−1^)^19^,^20^, and Bi_0.5_Sb_1.5_Te_3_ (~240 μV K^−1^)^21^. However, thanks to the significantly enhanced *σ* after doping, the power factor (*PF*) of the Cu_2_Sn_1−*x*_Zn_*x*_S_3_ (*x* = 0–0.20) samples increase largely with increasing *x*. The highest *PF* of ~0.84 mW m^−1^ K^−2^ at 723 K is achieved in the *x* = 0.20 sample, which is almost 20 times larger than that of the pristine CTS (0.047 mW m^−1^ K^−2^), and is comparable to that of Cu_2−*x*_S.

For further information about the effect of crystal structure on electrical transport properties, density-of-states (DOS) effective mass of carriers (m^*^) were calculated using the equation based on simple parabolic approximation^22^: 
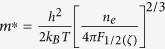
, where *h*, *k*_*B*_, *n*_*e*_, *F*_*n*_ and *ζ* are the Plank constant, Boltzmann constant, carrier concentration, Fermi integral, and chemical potential, respectively (see Supplementary Table S1). The obtained high m^*^ values (2.4–3.0) *m*_0_ with a high hole concentration level of 10^21^ cm^−3^ for samples with *x* = (0.10–0.20) are satisfactorily close, suggesting the minor influence of the two different crystal symmetries (cubic and tetragonal) on electronic structures. They are similar to the simulated single parabolic band model results of (2.5–4.2) *m*_0_ which correspond to carrier concentrations at 10^20^ cm^−3^ level^13^. The m^*^ value increases with *x*, from 1.45 *m*_0_ at 300 K for the *x *= 0.05 compound to 2.45 *m*_0_ for the *x *= 0.20 sample, close to the reported results in Zn-doped Cu_2_SnSe_3_^23^, and this behavior is qualitatively in accordance with the predicted higher m^*^ at elevated carrier concentrations for Cu_2_SnX_3_ (X = S, Se)^13^.

To clarify the physical mechanism for the increase of m^*^ with *x* in these samples, first principles calculations were performed and the DOS effective mass is calculated by fitting the calculated total DOS using parabolic bands. Results (see Supplementary Figure S1) indicate that the valance electronic structures after heavy Zn-doping are not changed substantially except for a shift of Fermi energy into deeper levels with larger *x*, which reflects the minor influence of acceptor dopant on the band structure of CTS. (Therefore in the following calculations, the DOS of non-doped Cu_2_SnS_3_ unit cell were used to calculate the m^*^ with the Fermi energy determined according to the measured carrier concentration.) As can be seen clearly in Table 2, the calculated m^*^ values increase gradually with the increase of *n* and are well in accordance with the results derived from measured Seebeck coefficients. Thus, it confirms that the increase of m^*^ drives from the Fermi energy shifting into deeper inside the valance band where the multiple hole pockets along the high-symmetry line A-M^13^ can also be incorporated, while without much effect due to the crystal structure evolution. This would be beneficial for attaining high power factor even at heavy Zn-doping levels.

**Figure 6:**
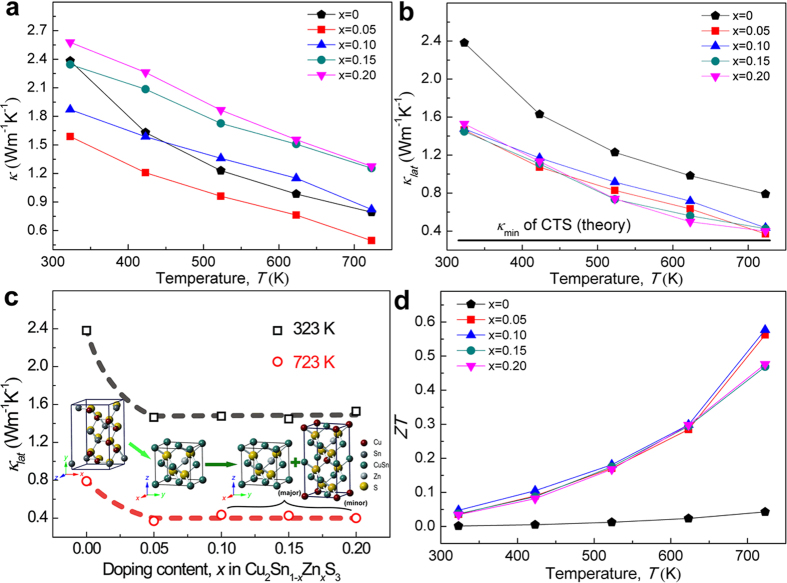
TE properties of the Cu_2_Sn_1−*x*_Zn_*x*_S_3_ samples: (**a**) thermal conductivity, (**b**) lattice thermal conductivity, (**c**) variation of lattice thermal conductivity with *x* at 323 K (black diamonds) and 723 K (red circles), respectively, with a schematic crystal structure evolution from monoclinic, cubic to coexistent phases, which are exactly composed of cubic (major) and tetragonal (minor) for Cu_2_Sn_1−*x*_Zn_*x*_S_3_. Dotted lines are imaginary tendency of *κ*_*lat*_ as a function of doping content, and (**d**) *ZT*. The theoretical minimum thermal conductivity of CTS at high temperatures (cited from ref. 13) is also plotted in (**b**) for comparison.

Thermal conductivities (*κ*) at 323–723 K for all samples are plotted in Fig. 6a. Generally, the *κ* values decrease largely from (2.1 ± 0.5) W m^−1^ K^−1^ at 323 K to a low level of (0.9 ± 0.4) W m^−1^ K^−1^ at 723 K, basically falling in a range for chalcogenide materials reported previously^21^,^24^,^25^,^26^,^27^. For a detailed inspection into the heat transport behavior, the electronic thermal conductivity was evaluated following the Wiedemann-Franz relation^28^ (*κ*_*e*_ = *LσT*, where the calculated Lorenz number, *L*, are given in Supplementary Figure S2), and the lattice component *κ*_*lat*_ was obtained by subtraction of the total *κ* with *κ*_*e*_. As shown in Fig. 6b, all the *κ*_*lat*_ values as a whole are relatively low (within 1.45–2.38 W m^−1^ K^−1^ at 323 K), as a primary result of the soft and inhomogeneous interatomic (Cu-S and Sn-S) bonding with different electronic and mass polarity. Approximately, they decrease proportionally to *T*^−1^ with temperature, implying the phonon-phonon Umklapp process dominating in the phonon transport.

More importantly, it shows obviously a large decrease in the *κ*_*lat*_ for the doped samples from that of pristine CTS in the whole temperature range, even approaching the theoretical minimum (0.3 W m^−1^ K^−1^) for Cu-Sn-S systems at high temperatures^13^. Meanwhile, for the doped samples, the *κ*_*lat*_ values are almost constant despite of their different doping levels, as shown in Fig. 6c. It has been known that with the structural change from pristine monoclinic into the cubic and tetragonal structures of doped samples, the local symmetry of [MS_4_] are improved while the metal atoms are rearranged from an ordered state in the monoclinic structure into a completely disordered state (at the 4*a* sites) in cubic structure and partially disordered state (at the 2*b* and 4*d* sites) in tetragonal structure according to the reported crystallographic database. Thus it concludes, the random (either completely or partially) distribution of Cu, Zn and Sn and vacancies, rather than the local distortion of [MS_4_] tetrahedra, may have played an important role in suppressing the lattice thermal conduction possibly by thoroughly disrupting the normal phonon transport^5^. In addition, the coexistence of cubic and tetragonal and even monoclinic phases should be taken into account of phonon scattering at the interfaces and grain boundaries.

The calculated dimensionless figure of merit (*ZT*) of Cu_2_Sn_1−*x*_Zn_*x*_S_3_ is shown in Fig. 6d. The *ZT*s increase with temperature in all samples. Though the largest *PF* = 0.84 mW m^−1^ K^−2^ is obtained for the *x* = 0.20 sample, its *ZT* suffers from the rise of *κ* by the large electronic contribution. Instead, the highest *ZT* ~ 0.58 is achieved in the *x* = 0.10 sample, which stands the highest among Pb-free sulfide TE materials except the PLEC Cu_2_S^3^ till now, and even surpasses many selenides and tellurides (e.g., Cu_1.8_S^2^ and CuGaTe_2_^29^) at the same temperature (see Supplementary Table S2).

## Conclusion

Cu_2_SnS_3_ (CTS) compounds can be viewed as a good thermoelectric material with structural and electrical features of phonon-glass-electron-structure, and can be tuned into excellent *p–*type TE materials through Zn-doping. First of all, CTS is favored by the disordered arrangement of metal atoms in the cubic and tetragonal phases and the interfaces and grain boundaries in the multiphase samples which significantly enhance the phonon scattering, leading to an ultralow lattice thermal conductivity. Second, CTS is benefited from its special electronic nature that is mainly determined by the Cu-3*d* and S-3*p* orbitals which is less affected by the coexistence of cubic and tetragonal phases, and allows relatively independent manipulation of carrier concentration through doping without affecting the valence band, and meanwhile, the DOS m^*^ that increases with carrier concentration ensures high power factor achievable by heavy doping. These findings are expected to be helpful for further investigation into the CTS-like compounds as environment-friendly TE materials. Based on the result that the lattice thermal conductivity is readily low at high temperatures, further efforts should be directed to the enhancement of *PF*, e.g. by way of band engineering or carrier energy filtering.

## Methods

Pristine and heavily hole-doped (by Sn-site-Zn-doping) CTS ingots with nominal compositions of Cu_2_Sn_1−*x*_Zn_*x*_S_3_ (*x* = 0, 0.05, 0.10, 0.15, 0.20) were first synthesized by direct reaction of high purity element powders with a molar ratio of Cu:Sn:Zn:S = 2:1−*x*:*x*:3 in a sealed silica tube at high temperatures. Powder obtained through grinding the corresponding ingot was then consolidated into pellets by spark plasma sintering under 50 MPa at 773 K for 5 min. Relative density of the as-produced pellets was determined by the Archimedes method to be ~94% of the theoretical value for all samples.

Phase composition and crystal structure were checked by X-ray diffraction (XRD) analysis with an ARL X’TRA diffractometer (SmartLab3, RIGAKU, Japan) using Cu Kα radiation. TEM specimens were prepared by a standard procedure which includes mechanical grinding, polishing, precision dimpling, and ion milling. SAED analyses and HRTEM observations were performed on an electron microscope operated at 200 kV (JEM-2100, JEOL, Japan). Seebeck coefficient and electrical conductivity were measured in the radial direction of a bar-shaped sample with dimensions of 10 mm × 3 mm × 3 mm by a conventional steady state method and a four-probe method, respectively, in a He atmosphere at 323–723 K with a commercial system (LSR-3). Hall effect measurement for carrier concentration and mobility was conducted with a van der Pauw configuration under vacuum using the ResiTest8300 system (Toyo Tech. Co.) Thermal diffusivity (*D*) was measured in the axial direction of a disk-shaped sample of Φ 10 mm × 1 mm using a Netzsch laser flash diffusivity instrument (LFA457, Netzsch, Germany). Thermal conductivity (*κ*) was calculated by *κ* = *DdC*_*p*_, where *C*_*p*_ is the specific heat capacity measured by differential scanning calorimetry (DSC: 2910, TA instruments), and *d* is the mass density measured using the Archimedes method.

The first principles calculations were performed using the Quantum-ESPRESSO package^30^, and the Garrity-Bennett-Rabe-Vanderbilt (GBRV) high-throughput pseudo-potential library^31^. Local density approximation (LDA) was used along with ultrasoft pseudopotentials for all the atoms. A plane wave basis with kinetic energy cutoff of 600 eV was used to ensure the convergence in all the calculations. A *k*-point sampling of 3 × 5 × 7 was used for Cu_2_SnS_3_ unit cell, and for larger cells the *k*-mesh are reduced accordingly. The atomic positions are relaxed until all the force components on each atom are less than 10^−3^ atomic unit. For density of states calculations, a denser *k*-point sampling of 5 × 7 × 11 was used for Cu_2_SnS_3_ unit cell.

## Additional Information

**How to cite this article**: Shen, Y. *et al*. Eco-friendly *p*-type Cu_2_SnS_3_ thermoelectric material: crystal structure and transport properties. *Sci. Rep.* **6**, 32501; doi: 10.1038/srep32501 (2016).

## Supplementary Material

Supplementary Information

## Figures and Tables

**Figure 1:**
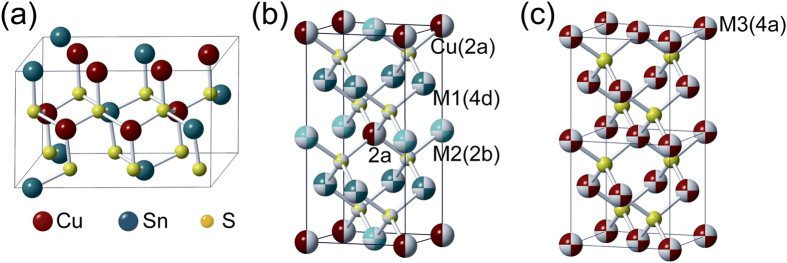
Illustration of (**a**) monoclinic, (**b**) tetragonal and (**c**) cubic crystal structures adopted by CTS with different metal arrangment.

**Table 1:** Crystal structure parameters of Cu_2_Sn_1−*x*_Zn_*x*_S_3_ (*x* = 0.10, 0.15 and 0.20) obtained from refinement of XRD.

*x*	Mole mass ratio	Lattice parameter (Å)			*R*_wp_ (%)
		*a*	*b*	*c*	
0.10	*F-43* *m* (54.8%)	5.432	5.432	5.432	8.17
	*I-42* *m* (24.1%)	5.493	5.493	11.010	
	*Cc* (21.1%)	6.622	11.509	6.477	
0.15	*F-43* *m* (33.8%)	5.432	5.432	5.432	9.93
	*I-42* *m* (60.9%)	5.710	5.710	10.260	
	*Cc* (5.3%)	6.208	13.550	6.955	
0.20	*F-43* *m* (34.4%)	5.445	5.445	5.445	6.56
	*I-42* *m* (60.6%)	5.736	5.736	10.273	
	*Cc* (4.9%)	6.237	13.571	7.003	

**Table 2:** Calculated DOS effective mass (*m* ^*^).

Fermi energy (eV)	Carrier concentration (10^21^ cm^−3^)	Calculated m^*^	m^*^ derived from measured Seebeck coefficients
−0.08	0.225	1.69	1.45
−0.25	1.96	2.29	2.39
−0.28	2.48	2.38	2.45

The Fermi energy is with respect to valance band maximum.
