# Supplementary material for: Eco-friendly p-type Cu2SnS3 thermoelectric material: crystal structure and transport properties
Source: Sci Rep. 2016 Sep 26;6:32501. doi: 10.1038/srep32501 (PMC5036087; doi:10.1038/srep32501)
Supplement: Supplementary Information [file srep32501-s1.pdf]

Eco-friendly *p*-type Cu_2SnS_3 thermoelectric material: crystal structure and transport properties

Yawei Shen[1], Chao Li[2], Rong Huang[2], Ruoming Tian[3], Yang Ye[1], Lin Pan[1,*], Kunihito Koumoto[3], Ruizhi Zhang[4], Chunlei Wan[5], Yifeng Wang[1,*]

[1]Nanjing Tech University, College of Materials Science and Engineering, Nanjing 210009, China

[2]East China Normal University, Key Laboratory of Polar Materials and Devices, Shanghai 200062, China

[3]Toyota Physical and Chemical Research Institute, Nagakute 480-1192, Japan

[4]Northwest University, Department of Physics, Xi'an 710069, China

[5]Tsinghua University, School of Materials Science and Engineering, Beijing 100084, China

[*]Corresponding author:

Tel: +86-159-5186-0386;

Fax: +86-25-83172261

E-mail: yifeng.wang@njtech.edu.cn (Y. Wang)

Tel: +86-182-5191-7814

E-mail: linpan@njtech.edu.cn (L. Pan)

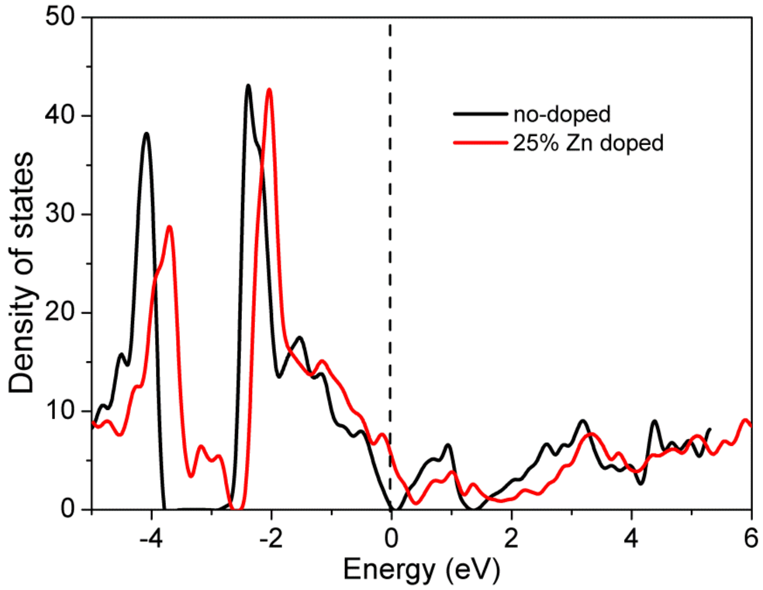

Figure S1. Total density of states of non-doped Cu_2SnS_3 unit cell and 25% mol Zn doping supercell. The Fermi energy was set to 0.

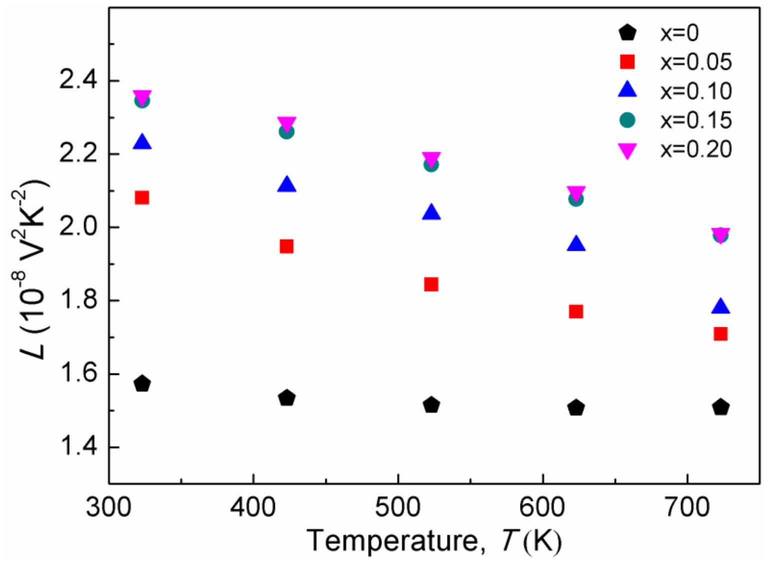

Figure S2. Calculated temperature-dependent Lorenz number for $Cu_2Sn_{1-x}Zn_xS_3$ samples.

To assume a single parabolic band dominated by acoustic phonon scattering, the Lorenz number, *L*, can be obtained by fitting the Seebeck coefficient to the reduced chemical potential: [1 2]

$$L = \left(\frac{k_B}{e}\right)^2 \frac{3F_0(\eta)F_2(\eta) - 4F_1(\eta)^2}{F_0(\eta)^2} \quad (1)$$

$$S = \frac{k_B}{e}\left(\frac{2F_1(\eta)}{F_0(\eta)} - \eta\right) \quad (2)$$

$$F_n(\eta) = \int_0^\infty \frac{\chi^n}{1+e^{\chi-\eta}}\, d\chi \quad (3)$$

Where k_B is the Boltzmann constant, and e is the electron charge, η is the reduced Fermi energy, $F_n(\eta)$ is the n-th order Fermi integral. The calculated values of L are shown in **Figure S2**.

**T (K)**	**Chemical potential ζ (eV)**			**Carrier concentration n_e (10^{21} cm^{-3})**			**m* derived from measured Seebeck coefficients**		
	x=0.10	0.15	0.20	x=0.10	0.15	0.20	x=0.10	0.15	0.20
300	6.98	9.52	10.35	1.30	2.08	2.50	2.35	2.39	2.45
373	5.65	8.70	9.31	1.35	2.15	2.54	2.37	2.54	2.67
523	3.39	4.80	5.05	1.30	2.09	2.37	2.63	2.64	2.74
673	2.33	3.32	3.43	1.25	2.04	2.38	2.69	2.80	3.03

Table S1. Calculated DOS effective mass (m*). The Fermi energy is with respect to valance band maximum.

Samples	Conduction type	ZT value	Temperature	Ref.
$Cu_2Sn_{0.9}Zn_{0.1}S_3$	p	0.58	723 K	This work
$Cu_{1.8}S$	p	0.30	673 K	[3]
$Cu_{1.8}S + Cu_{1.96}S$	p	0.50	673 K	[3]
$Cu_{1.97}S$	p	0.97	723 K	[4]
$Cu_4Mo_6S_8$	p	0.28	700 K	[5]
$Cu_{2.1}Zn_{0.9}SnS_4$	p	0.38	700 K	[6]
Bi_2S_3	n	0.22	573 K	[7]
$Ti_{0.95}Ta_{0.05}S_2$	n	0.28	700 K	[8]
$(SnS)_{1.2}(TiS_2)_2$	n	0.37	723 K	[9]
PbS	n	0.50	723 K	[2]
$PbS_{1-x}Te_x$ + 0.1% $PbCl_2$	n	0.67	723 K	[2]
$Cu_{0.97}Fe_{1.03}S_2$	n	0.33	700 K	[10]
$NdGd_{1.01}S_3$	n	0.32	723 K	[11]
$Cu_{1.975}Sn_{1.025}Se_3$	p	0.34	700 K	[12]
$Cu_{2.1}Zn_{0.9}SnSe_4$	p	0.45	700 K	[6]
$Cu_2Sn_{0.925}In_{0.075}S_{0.9}Se_{2.1}$	p	0.53	723 K	[13]
$Cu_2ZnSn_{0.9}In_{0.1}Se_4$	p	0.40	723 K	[14]
$CuGaTe_2$	p	0.50	723 K	[15]
$Cu_2Ga_4Te_7$	p	0.30	723 K	[16]
$Cu_2In_4Te_7$	p	0.30	700 K	[17]
$Ni_{0.05}Mo_3Sb_{5.4}Te_{1.6}$	p	0.25	700 K	[18]

Table S2 Conduction type and ZT value for present $Cu_2Sn_{0.9}Zn_{0.1}S_3$ material and other typical chalcogenide thermoelectric materials.

References

1. May, A. F., Toberer, E. S., Saramat, A. & Snyder, G. J. Characterization and analysis of thermoelectric transport in n-type $Ba_8Ga_{16-x}Ge_{30+x}$. *Phys. Rev. B* **80**, 125205 (2009).

2. Johnsen, S. *et al.* Nanostructures boost the thermoelectric performance of PbS. *J. Am. Chem. Soc.* **133**, 3460-3470 (2011).

3. Ge, Z. H. *et al.* Synthesis and transport property of $Cu_{1.8}S$ as a promising thermoelectric compound. *Chem. Commun.* **47**, 12697-12699 (2011).

4. He, Y. *et al.* High thermoelectric performance in non-toxic earth-abundant copper sulfide. *Adv. Mater.* **26**, 3974-3978 (2014).

5. Ohta, M., Obara, H. & Yamamoto, A. Preparation and Thermoelectric Properties of Chevrel-Phase $Cu_xMo_6S_8$ ($2.0 \leq x \leq 4.0$). *Mater. Trans.* **50**, 2129-2133, (2009).

6. Liu, M.-L., Huang, F.-Q., Chen, L.-D. & Chen, I. W. A wide-band-gap p-type thermoelectric material based on quaternary chalcogenides of Cu_2ZnSnQ_4 (Q = S, Se). *Appl. Phys. Lett.* **94**, 202103 (2009).

7. Ge, Z.-H. *et al.* Enhancing thermoelectric properties of polycrystalline Bi_2S_3 by optimizing a ball-milling process. *J. Electron. Mater.* **40**, 1087-1094 (2011).

8. Beaumale, M. *et al.* Thermoelectric properties in the series $Ti_{1-x}Ta_xS_2$. *J. Appl. Phys.* **115**, 043704 (2014).

9. Wan, C., Wang, Y., Wang, N. & Koumoto, K. Low-thermal-conductivity $(MS)_{1+x}(TiS_2)_2$ (M = Pb, Bi, Sn) misfit layer compounds for bulk thermoelectric materials. *Materials* **3**, 2606-2617 (2010).

10. Li, Y. *et al.* Thermoelectric transport properties of diamond-like $Cu_{1-x}Fe_{1+x}S_2$ tetrahedral

compounds. *J. Appl. Phys.* **116**, 203705 (2014).

11. Ohta, M. & Hirai, S. Thermoelectric properties of $NdGd_{1+x}S_3$ prepared by CS_2 sulfurization. *J. Electron. Mater.* **38**, 1287-1292 (2009).

12. Fan, J. *et al.* Structural evolvement and thermoelectric properties of $Cu_{3-x}Sn_xSe_3$ compounds with diamond-like crystal structures. *Dalton T.* **43**, 16788-16794 (2014).

13. Skoug, E. J., Cain, J. D. & Morelli, D. T. Improved thermoelectric performance in Cu-based ternary chalcogenides using S for Se substitution. *J. Electron. Mater.* **41**, 1232-1236 (2012).

14. Shi, X. Y., Huang, F. Q., Liu, M. L. & Chen, L. D. Thermoelectric properties of tetrahedrally bonded wide-gap stannite compounds $Cu_2ZnSn_{1-x}In_xSe_4$. *Appl. Phys. Lett.* **94**, 122103 (2009).

15. Plirdpring, T. *et al.* Chalcopyrite $CuGaTe_2$: a high-efficiency bulk thermoelectric material. *Adv. Mater.* **24**, 3622-3626 (2012).

16. Plirdpring, T. *et al.* High-temperature thermoelectric properties of $Cu_2Ga_4Te_7$ with defect zinc-blende structure. *Appl. Phys. Lett.* **98**, 172104 (2011).

17. Plirdpring, T. *et al.* High-temperature thermoelectric properties of $Cu_2In_4Te_7$. *Phys. Status Solidi – R.* **6**, 154–156 (2012).

18. Nandihalli, N. *et al.* Thermoelectric properties of composites made of $Ni_{0.05}Mo_3Sb_{5.4}Te_{1.6}$ and fullerene. *J. Solid State Chem.* **203**, 25-30 (2013).
